# Participation in Social Activities among Adolescents with an Autism Spectrum Disorder

**DOI:** 10.1371/journal.pone.0027176

**Published:** 2011-11-14

**Authors:** Paul T. Shattuck, Gael I. Orsmond, Mary Wagner, Benjamin P. Cooper

**Affiliations:** 1 Brown School of Social Work, Washington University, St. Louis, Missouri, United States of America; 2 Department of Occupational Therapy at Boston University, Boston, Massachusetts, United States of America; 3 SRI International, Menlo Park, California, United States of America; French National Centre for Scientific Research, France

## Abstract

**Background:**

Little is known about patterns of participation in social activities among adolescents with an autism spectrum disorder (ASD). The objectives were to report nationally representative (U.S.) estimates of participation in social activities among adolescents with an ASD, to compare these estimates to other groups of adolescents with disabilities, and examine correlates of limited social participation.

**Methods and Findings:**

We analyzed data from wave 1 of the National Longitudinal Transition Study 2, a large cohort study of adolescents enrolled in special education. Three comparison groups included adolescents with learning disabilities, mental retardation, and speech/language impairments. Adolescents with an ASD were significantly more likely never to see friends out of school (43.3%), never to get called by friends (54.4%), and never to be invited to social activities (50.4%) when compared with adolescents from all the other groups. Correlates of limited social participation included low family income and having impairments in conversational ability, social communication, and functional cognitive skills.

**Conclusions:**

Compared with prior research, our study significantly expands inquiry in this area by broadening the range of social participation indicators examined, increasing the external validity of findings, focusing on the under-studied developmental stage of adolescence, and taking an ecological approach that included many potential correlates of social participation. There were notable differences in social participation by income, a dimension of social context seldom examined in research on ASDs.

## Introduction

A growing number of children identified with an autism spectrum disorder (ASD) are aging into adolescence and toward adulthood. Recent estimates place the prevalence of ASD at 1 in 110 children [1]. However, very little is known about the course of ASD through adolescence and into young adulthood. Difficulty with social interaction is a defining feature of ASDs [2]. As the demands for and complexity of social interactions increase during adolescence, teens with an ASD face significant difficulty navigating peer relationships. Yet, there is relatively little research describing their participation in social activities. Limited or absent peer relationships can negatively influence health and mental health, especially during adolescence [3].

Focusing on the social participation of adolescents is consistent with the World Health Organization's 2002 International Classification of Functioning, Disability, and Health, which describes the outcomes of disability as a function of both individual characteristics and contextual factors [4]. To date, evidence describing social participation in adolescents with an ASD has focused primarily on the arena of friendships, and has typically been conducted with small, select samples [5]. One prior large-scale study describing friendships in both children and adolescents with an ASD has been published, and it was based on one item from the Autism Diagnostic Interview-Revised, for a sample of 1,202 children and adolescents (ages 4 to 17 years) with an ASD whose families participated in the Simons Simplex Collection [6]. These researchers found that 24.3% of participants had no reciprocal peer relationships. Results were not stratified by age, so we do not have current estimates of friendship specific to adolescence. A study of youth with an ASD ages 17 to 21 years found that 55.4% had never gotten together with a friend and 63.9% had never been called on the phone by a friend in the 12 months prior to the survey [7].

A fuller, more nuanced understanding of the prevalence and correlates of social participation among adolescents with an ASD can support the development and provision of appropriate services to this vulnerable and growing group in several ways. Identifying subgroups of adolescents with an elevated risk of limited social participation can help inform decisions about targeting finite resources to aid those with the highest need. Testing for disparities by demographic factors like race and income can suggest a need for policies aimed at reaching out to underserved subpopulations. Understanding more about the correlates of social participation can help suggest person-level intervention targets that might offer the most leverage for improving outcomes. Lastly, answers to these questions can help increase the awareness of clinicians and trainees regarding the broader social context and challenges of their patient population.

In this study, we used data from a large nationally representative cohort study to estimate social participation rates among adolescents with an ASD to address the following research questions. What are the rates of participation in social activities among adolescents with an ASD? How do these participation rates compare to adolescents with other kinds of disabilities? And finally, what are the correlates of limited participation? To contextualize findings, adolescents with an ASD were compared with three groups of peers likely to have impairments in some, but not all, of the areas of development affected by ASD: those with intellectual disabilities, speech impairments, or learning disabilities.

One recent study used the same data set to examine similar questions [7] However, the findings from the two studies are not directly comparable. Our study focused on social participation using data from the first wave of the cohort study when participants were all younger than 18 years old and still in high school. The other article focused on a subset of participants who still remained in the study at a later wave of data collection when students were 17 to 21 years of age, and included some who were still in high school and others who no longer were in high school. Furthermore, we examined a larger and more diverse set of social participation outcomes and correlates.

Answering the questions we pose will increase awareness of social participation limitations in this population. National prevalence estimates can provide service providers and policy-makers with benchmark information for contextualizing local estimates and whether change is occurring over time. Provision and coordination of out-of-school activities is left largely to parental initiative. By describing current participation patterns, identifying subgroups of adolescents with an ASD who are more restricted in their participation, and identifying the correlates of limited participation, we will be able to better target the needs of this vulnerable population and potentially improve their quality of life.

## Methods

### Ethics Statement

Use of these data is governed by a data-use agreement with the U.S. Department of Education and was deemed exempt by the Washington University Institutional Review Board.

### Study Sample

The National Longitudinal Transition Study-2 (NLTS2) was a prospective study conducted by SRI International for the U.S. Department of Education with data collected from parents and/or adolescents in five waves, 2 years apart, from 2001 to 2009. The study sampled about 11,000 adolescents receiving special education services at baseline and followed them as they aged into young adulthood. This paper is based on data from wave 1, collected in 2001. Unweighted sample sizes in this report were rounded to the nearest ten, as required by the U.S. Department of Education.

The NLTS2 sampling plan was designed to yield nationally representative estimates that would generalize to all students receiving special education services who were in 7^th^ through 12^th^ grade or in ungraded programs and who were ages 13 through 16 on December 1, 2000. The multistage sampling procedure sampled school districts first and then students within districts [8]. There are unique analysis weights for each instrument and each wave of data collection so that estimates generalize to the national population of youth who were receiving special education services in a given age range and disability type. Full details of the weighting strategy for NLTS2 were previously published [8].

For the sake of official special education enrollment reports, each student is counted only once in a primary disability category. Autism is one of thirteen primary disability categories mandated by the Individuals with Disabilities Education Act. Diagnostic criteria from the *Diagnostic and Statistical Manual of Mental Disorders* (Fourth Edition) (*DSM-IV*) are not necessarily used by schools for special education eligibility determination and enrollment classification [9]. The definition of autism by the U.S. Department of Education is less detailed than but nonetheless consistent with *DSM-IV* criteria. Population-based research in the U.S. has consistently found that the vast majority (>95%) of children receiving special education services in the autism category also meet *DSM-IV*-based case criteria for an ASD [10], [11]. These reports suggest the classification of children into the special education autism category is moderately sensitive and very specific. Although not all adolescents with an ASD are served via the special education autism designation, it is unlikely that students enrolled in this category do not have an ASD. An unknown proportion of adolescents with an ASD participate in special education, but via other eligibility categories, such as mental retardation.

Each student's eligibility for special education services was determined by the school district from whose roster the student was sampled. There is some unknown amount of inter-district variability in eligibility criteria. In the NLTS2 data set, the number of sample-eligible students in the autism category was 1,100. There were 920 participants with parent interview data at wave 1, for a response rate of 84%. We restricted analyses to adolescents who were in school during the prior year because several outcomes we examined related to school-based activities, reducing the number to 900.

We conducted descriptive comparisons of the prevalence of social activities between adolescents in the autism category and adolescents from three other special education disability categories: speech/language (SP) impairment, learning disability (LD), and mental retardation (MR). Current consensus in the field eschews use of the term “mental retardation” in favor of “intellectual disability” [12]. However, we use the term “MR” to be consistent with the special education legislative definitions of the various disability categories and the way the survey data were collected. We excluded 30 comparison group members from analyses who also had a parent report of ever receiving an autism-related diagnosis.

### Data collection procedures

This study draws on data from three sources. First, parent/guardian telephone interviews were conducted in 2001. The interview began by identifying the adult who was best able to respond about the sampled youth; 91% of respondents for ASD adolescents were parents. An abbreviated mail questionnaire was sent to 30 ASD families (3%) who were unavailable by telephone. Second, for each school attended by an NLTS2 sample member, a school staff person knowledgeable about the characteristics and policies of those schools (often the principal) was surveyed by mail. Broad information about the school and the student body was collected. School-level information was linked to each NLTS2 sample member enrolled at a given school. Third, a survey was mailed to the school staff member most familiar with each student's school program, often a special educator.

### Measures and variables

We examined 13 measures of social activity and participation divided into three categories: social participation with friends, general social participation, and disability-related social participation (described in detail in Table 1). We used dichotomous indicators of limited social participation in four logistic regression models: never sees friends, friends never call, never invited to activities, no extracurricular activities. The latter two were dichotomous to begin with. The former two were recoded from four-category ordinal questionnaire responses. We used this strategy to facilitate interpreting all four models consistently as correlates of the complete absence of social participation, an unfortunately common outcome among adolescents with an ASD.

**Table 1:** Outcome measures used in the study.

Measure	Description
**Social Participation with Friends**	
Sees friends	Parents were asked about the frequency their son/daughter got together with friends outside of school or organized activities during the past 12 months. The “never” category was used for logistic regression.
Friends call	During the prior 12 months, how often friends have called by phone. The “never” category was used for logistic regression.
Invited to activities	During the prior 12 months, whether invited by friends to any social activity. The “never” category was used for logistic regression.
**General Social Participation**	
Performs volunteer or community service	Any volunteer or community service activities in the prior 12 months.
Take lessons or classes outside of school	Any classes or lessons outside of school in the prior 12 months (e.g., art, music, computers).
Has any nonschool activities	Any nonschool group activities during the prior 12 months (e.g., scouting, church youth group, nonschool sports teams).
Has any school activity other than class	Any school activity outside of class in the prior 12 months (e.g., band, sports, student government, clubs).
Has any extracurricular activities	If youth participated in any of the 4 types of activities listed above.
Kinds of groups youth belonged to	A subset of respondents were asked an open-ended follow up question about types of groups each youth belonged to in the past year if previous questions indicated that the youth had participated in any activities. We examined the groups with a large enough cell size to make data analysis feasible: religious youth group, sports team, performing group. Adolescents with no participation were coded to 0 so the denominator included all adolescents. Thus, the point estimates represent the population prevalence of group participation rather than the subpopulation estimate of participation among those with at least some participation.
Count of typical groups	Count variable created from 11 groups that are socially oriented: scouting, religious group, YMCA/JCC/etc., sports, special interest club, performing group, student government, subject club, volunteer service, cultural affinity, leadership. Excluded were disability-specific, academically remedial, or vocationally-oriented activity groups.
**Disability-Related Social Participation**	
Special needs group	Belonged to any group in the past year that only includes youth with special needs (e.g., Special Olympics, disability support group).

Covariates included demographic factors, behavioral characteristics, family socioeconomic resources, and school characteristics. We included student ethnicity and race to be able to identify disparities related to those factors. We included an indicator for parent-reported diagnosis of attention deficit hyperactivity disorder (ADHD) because it is a common comorbidity among those with an ASD [13]. Unfortunately, the survey did not directly ask parents about other common comorbidities such as intellectual disability.

A scale of externalizing behaviors was created by summing five 3-category (never, sometimes, very often) ordinal measures of how often each youth: ended disagreements calmly (reverse coded), behaved at home in a way that caused problems for the family, received criticism well (reverse coded), controlled temper when arguing (reverse coded), and got into situations resulting in trouble (Cronbach's alpha  = .60 in the ASD group). A 4-category ordinal question asked parents how well their child could carry on a conversation. A scale of social communication behaviors was created by summing four 3-category (never, sometimes, very often) ordinal measures of how often each youth: joined group activities without being told to, made friends easily, seemed confident in social situations such as parties or group outings, and started conversations rather than waiting for others to start (could include sign language and other means of communication) (Cronbach's alpha  = .74 in the ASD group). A functional cognitive skills scale was constructed by summing four 4-category (not at all well, not very well, pretty well, very well) questions about how well a youth could do the following tasks without help: tell time on an analog clock, read and understand common signs, count change, and look up telephone numbers and use a telephone (Cronbach's alpha  = .87 in the ASD group).

We examined three school and program factors that might influence social participation. Schools were grouped into three types: regular, special (i.e., serving only students with disabilities), and other (e.g., charter schools, magnet schools). School size measured the number of students attending a given youth's school, and an indicator measured whether each student spent any part of their day in a special education classroom.

### Data Analysis

Rates of missing data per variable for parent interview items ranged from 0% to 16%, with one variable missing more than 6% (income: 16%) and the remaining variables missing less than 6%. Missing rates were higher for variables from the student program survey (any part of the day spent in special education class: 52%) and school characteristics survey (school type: 24%, number of enrolled students: 25%) due to lower response rates on those instruments. Missing data were imputed using sequential regression in IVEware (version 0.1) to create 50 sets of data with no missing values [14], [15].

All reported estimates were weighted and variances adjusted to account for the complex sampling and the multiple imputation using the “mi svy” procedures available in Stata v 11, which uses standard methods for combining estimates in the analysis of multiply imputed data [16]. Univariate point estimates and 95% confidence intervals were computed for describing the independent variables (Table 2). Rates of social participation were compared across groups (Table 3). Crosstabulation tests are not available in Stata for multiply imputed data. Therefore, we tested for the significance of differences between the adolescents with ASD vs. each other group of adolescents using logistic regression with dummy coding. Confidence intervals were omitted from this table for ease of reading but are available from the corresponding author.

**Table 2:** Distributions of covariates within each group [95% confidence intervals].

	Autism Spectrum Disorder	Speech-Language Impairment	Learning Disability	Mental Retardation
**Age**				
13 & 14	33.0% [28.5, 37.8]	44.4% [39.8, 49.1]	31.5% [28.1, 35.0]	27.0% [23.5, 30.9]
15	22.9% [19.6, 26.5]	22.7% [19.9, 25.7]	24.2% [21.1, 27.5]	23.1% [19.9, 26.7]
16	26.3% [22.6, 30.4]	20.1% [16.3, 24.5]	26.0% [22.4, 29.9]	28.3% [24.9, 32.0]
17	17.8% [14.8, 21.4]	12.8% [10.5, 15.7]	18.4% [15.3, 22.0]	21.6% [18.4, 25.1]
**Female**	15.5% [13.0, 18.5]	37.8% [33.5, 42.3]	33.0% [29.4, 37.0]	43.1% [39.5, 46.7]
**Hispanic**	11.0% [7.3, 16.4]	19.1% [9.8, 33.7]	20.8% [15.9, 26.7]	10.9% [7.7, 15.3]
**Race**				
White	65.1% [59.4, 70.4]	65.1% [55.8, 73.4]	66.8% [59.7, 73.3]	57.4% [51.7, 63.0]
African- American	22.7% [17.8, 28.4]	16.5% [12.4, 21.5]	16.7% [12.7, 21.6]	32.2% [27.1, 37.7]
Other	12.2% [9.4, 15.8]	18.4% [12.0, 27.2]	16.5% [12.8, 20.8]	10.4% [8.2, 13.1]
**Income**				
< $25,000	22.0% [18.0, 26.5]	26.4% [20.5, 33.3]	30.7% [26.0, 35.8]	50.6% [45.8, 55.4]
$25,001–50,000	31.9% [27.5, 36.7]	34.3% [30.0, 39.0]	33.5% [29.8, 37.4]	31.1% [27.0, 35.4]
$50,001–75,000	23.2% [19.6, 27.1]	23.7% [19.2, 28.9]	24.5% [20.3, 29.3]	11.8% [9.3, 14.8]
> $75,000	23.0% [18.7, 27.9]	15.5% [11.2, 21.1]	11.3% [8.7, 14.6]	6.6% [4.6, 9.2]
**Externalizing behaviors (mean)**	4.1 [3.9, 4.3]	3.4 [3.3, 3.5]	3.9 [3.7, 4.1]	4.2 [4.0, 4.3]
**How well youth converses**				
No trouble	13.4% [10.6, 16.7]	61.2% [55.2, 66.9]	75.8% [71.4, 79.6]	44.1% [40.3, 48.0]
A little trouble	31.1% [27.1, 35.3]	29.9% [25.9, 34.3]	21.0% [17.6, 24.9]	34.4% [31.0, 37.9]
A lot of trouble	38.8% [34.7, 43.1]	8.6% [6.0, 12.1]	3.2% [2.2, 4.7]^a^	17.0% [13.9, 20.6]
No conversation	16.7% [12.5, 22.1]	0.3% [0.1, 1.1]		4.6% [3.2, 6.6]
**Social communication (mean)**	2.9 [2.7, 3.1]	5.0 [4.8, 5.3]	5.3 [5.1, 5.6]	4.6 [4.5, 4.8]
**Functional cognitive skills (mean)**	10.9 [10.4, 11.4]	14.3 [14.0, 14.7]	14.0 [13.8, 14.3]	11.3 [11.0, 11.6]
**ADHD**	34.3% [29.9, 38.9]	19.2% [15.8, 23.0]	33.5% [29.4, 37.8]	29.9% [26.1, 34.0]
**Type of school**				
Regular	85.4% [79.7, 89.7]	95.2% [92.4, 97.1]	95.4% [92.4, 97.3]	93.8% [91.2, 95.7]
Special	10.4% [6.7, 15.9]	4.8% [2.9, 7.6]^b^	4.6% [2.7, 7.6]^b^	3.6% [2.2, 5.7]
Other	4.2% [2.6, 6.7]			2.7% [1.5, 4.5]
**School size (mean)**	1,354 [1,204,1,503]	1,377 [1,218, 1,536]	1,346 [1,254, 1,437]	1,153 [1,077, 1,229]
**Any part of day spent in special classroom**	88.2% [83.8, 91.6]	63.4% [56.7, 69.7]	73.2% [68.3, 77.6]	94.5% [90.8, 96.8]

Source: National Longitudinal Transition Study 2.

Notes: Number of multiply imputed data sets  = 50. Weighted to population levels. Variances adjusted for sampling method.

a The “A lot of trouble” and “No conversation” category estimates for ‘How well youth converses’ were combined for the Learning Disability group in compliance with U.S. Department of Education rules aimed at preventing data disclosure in instances where point estimates are based on very few underlying cases.

b The “Special” and “Other” category estimates for ‘Type of school’ were combined for the Speech-Language Impairment and Learning Disability groups in compliance with U.S. Department of Education rules aimed at preventing data disclosure in instances where point estimates are based on very few underlying cases.

**Table 3:** Rates (percentages unless otherwise noted) of social participation compared among groups, tests are for significant difference between each comparison group and the autism spectrum disorder group.

	Autism Spectrum Disorder	Speech-Language Impairment	Learning Disability	Mental Retardation
***Social Participation with Friends***				
**Sees friends**				
Never	43.3	8.7***	6.7***	15.7***
Sometimes, not weekly	32.1	23.0**	23.5**	29.6
About once weekly	10.0	12.3	11.4	12.1
> once weekly	14.6	56.1***	58.5***	42.7***
**Friends call**				
Never	54.4	5.7***	2.9***	16.9***
Less than monthly	19.8	8.0***	8.3***	14.5*
A few times per month	9.7	8.0	7.2	9.1
About once weekly	6.5	12.7**	10.0	11.9*
> once weekly	9.6	65.6***	71.6***	47.7***
**Invited to activities**	49.6	89.2***	88.7***	75.9***
***General Social Participation***				
Performs volunteer or community service	35.1	46.0***	43.0***	33.3
Take lessons or classes outside of school	30.6	28.4	23.6*	19.6***
Has any nonschool activities	43.8	54.5**	50.7*	40.9
Has any school activity other than class	30.2	58.5***	49.2***	34.0
Any extracurricular activities [yes if any of the 4 above items]	70.6	80.4***	78.2***	69.8
**Kinds of groups youth belonged to**				
Religious youth group	27.3	34.4*	32.7*	27.2
Sports team	16.3	41.0***	36.0***	22.3**
Performing group	9.7	20.2***	12.3	7.4
Mean count of typical groups (not%)	0.7	1.2***	1.0***	0.8
**Disability-Related Social Participation**				
Special needs group	24.9	2.8***	3.2***	15.4***

*p <.05,

**p <.01,

***p <.001.

Source: National Longitudinal Transition Study 2.

Notes: Number of multiply imputed data sets  = 50. Weighted to population levels. Variances adjusted for sampling method.

Within the ASD group, we stratified the rates of limited social participation by each of the correlates (Table 4). Logistic regression models estimated the adjusted association between correlates and the four indicators of limited social participation among the adolescents with ASD (Table 5).

**Table 4:** Percentages and 95% confidence intervals of limited social participation among adolescents with an autism spectrum disorder, overall and stratified by covariates.

	Never sees friends	Friends never call	Never invited to activities	No extracurricular activities
**Overall rate**	43.3 [38.8, 47.9]	54.4 [48.7, 59.9]	50.4 [44.8, 56.0]	29.4 [25.8, 33.3]
**Age**				
13 & 14	45.4 [38.4, 52.6]	57.3 [49.1, 65.0]	52.4 [44.1, 60.5]	33.5 [28.2, 39.4]
15	49.9 [41.1, 58.7]	57.5 [49.2, 65.5]	53.3 [44.1, 62.3]	28.1 [20.9, 36.6]
16	39.6 [31.5, 48.3]	53.5 [44.0, 62.8]	49.9 [40.7, 59.1]	30.8 [22.8, 40.1]
17	36.4 [25.8, 48.5]	46.2 [34.1, 58.7]	43.9 [33.0, 55.4]	21.4 [14.2, 31.0]
**Gender**				
Male	43.1 [38.0, 48.4]	54.8 [48.6, 60.8]	50.5 [44.8, 56.2]	29.5 [25.6, 33.6]
Female	44.2 [34.5, 54.4]	52.2 [41.5, 62.7]	49.9 [38.9, 61.0]	29.2 [21.7, 37.9]
**Ethnicity**				
Not Hispanic	41.6 [36.9, 46.5]	52.7 [47.3, 58.1]	50.4 [44.9, 55.9]	27.9 [24.2, 32.0]
Hispanic	56.8 [44.1, 68.7]	67.7 [52.8, 79.6]	50.5 [36.4, 64.6]	41.5 [31.7, 51.9]
**Race**				
White	41.6 [36.0, 47.6]	49.9 [42.9, 56.8]	48.8 [41.8, 55.9]	27.6 [22.6, 33.3]
African-American	44.3 [36.0, 53.0]	60.8 [52.4, 68.6]	51.5 [43.2, 59.6]	32.4 [25.8, 39.7]
Other	50.1 [38.1, 62.2]	66.5 [54.1, 76.9]	57.0 [43.1, 69.9]	33.5 [24.9, 43.4]
**Income**				
< $25,000	53.1 [43.7, 62.3]	71.7 [63.3, 78.9]	58.5 [48.7, 67.6]	43.2 [35.2, 51.5]
$25,001–50,000	45.9 [38.2, 53.8]	52.4 [43.4, 61.2]	51.6 [42.7, 60.5]	31.1 [24.3, 38.7]
$50,001–75,000	40.9 [31.6, 50.8]	50.2 [41.1, 59.3]	53.4 [44.4, 62.2]	23.0 [16.8, 30.7]
> $75,000	32.8 [25.5, 41.1]	44.7 [35.4, 54.4]	38.0 [30.1, 46.6]	20.5 [14.1, 28.8]
**Externalizing behaviors**				
< = mean	38.4 [33.5, 43.6]	50.4 [44.2, 56.6]	47.2 [41.3, 53.1]	27.8 [23.1, 33.0]
> mean	49.5 [42.1, 57.0]	59.5 [51.4, 67.1]	54.6 [46.6, 62.3]	31.5 [26.5, 37.1]
**How well youth converses**				
No trouble	22.0 [14.2, 32.2]	21.3 [13.4, 31.9]	20.9 [13.7, 30.5]	11.8 [7.2, 18.8]
A little trouble	29.2 [23.6, 35.5]	29.1 [23.4, 35.6]	42.3 [35.4, 49.6]	21.6 [16.5, 27.8]
A lot of trouble	55.0 [47.7, 62.0]	69.2 [61.9, 75.7]	58.3 [51.3, 65.0]	30.7 [25.5, 36.5]
No conversation	59.5 [47.5, 70.4]	93.3 [88.0, 96.4]	70.7 [58.5, 80.5]	55.0 [45.4, 64.2]
**Social communication**				
< = mean	69.1 [63.1, 74.4]	75.8 [69.4, 81.2]	73.2 [67.0, 78.7]	36.0 [30.9, 41.5]
> mean	21.5 [17.1, 26.5]	36.2 [30.0, 42.9]	31.1 [25.3, 37.6]	23.8 [19.3, 29.0]
**Functional cognitive skills**				
< = mean	53.3 [47.5, 59.0]	76.5 [70.9, 81.2]	61.7 [54.6, 68.2]	41.2 [36.1, 46.5]
> mean	35.0 [29.3, 41.2]	36.0 [30.2, 42.2]	41.1 [34.8, 47.6]	19.6 [15.7, 24.2]
**ADHD Status**				
No	44.8 [39.6, 50.1]	57.1 [50.7, 63.3]	52.6 [46.2, 59.0]	31.3 [27.1, 35.7]
Yes	40.4 [32.8, 48.5]	49.1 [40.8, 57.5]	46.2 [38.2, 54.3]	25.9 [20.0, 32.9]
**Type of school**				
Regular	41.9 [36.9, 47.0]	52.2 [46.4, 57.9]	49.2 [43.4, 54.9]	28.6 [24.7, 32.9]
Special	63.3 [53.4, 72.2]	77.8 [67.9, 85.4]	67.7 [57.5, 76.6]	41.5 [32.9, 50.7]
Other	23.0 [10.0, 43.2]	41.0 [20.5, 64.9]	33.5 [16.0, 56.6]	15.6 [5.3, 35.9]
**School size**				
< = mean	44.1 [37.5, 50.9]	58.3 [50.0, 66.2]	52.4 [44.8, 59.8]	28.2 [23.1, 33.8]
> mean	42.4 [35.8, 49.2]	49.7 [42.6, 56.8]	48.1 [40.8, 55.4]	30.9 [25.4, 37.0]
**Any part of day in special classroom**				
No	24.3 [14.0, 38.2]	27.1 [16.4, 41.0]	31.9 [19.7, 46.9]	21.6 [12.7, 33.7]
Yes	45.8 [41.1, 50.7]	58.0 [52.1, 63.6]	52.9 [47.0, 58.7]	30.5 [26.6, 34.6]

**Table 5:** Logistic regression models of factors associated with limited social participation among adolescents with an autism spectrum disorder (odds ratios and 95% confidence intervals, reference groups in parentheses).

	Never sees friends	Friends never call	Never invited to activities	No extracurricular activities
**Age**				
(13 & 14)	-	-	-	-
15	1.31 [0.77,2.21]	1.02 [0.58,1.79]	0.99 [0.62,1.57]	0.68 [0.43,1.07]
16	0.93 [0.58,1.50]	1.01 [0.62,1.65]	1.01 [0.62,1.64]	0.84 [0.49,1.44]
17	0.69 [0.36,1.33]	0.79 [0.42,1.49]	0.79 [0.43,1.44]	0.58 [0.33,1.00]
**Female**	0.84 [0.46,1.54]	0.53 [0.28,1.01]	0.74 [0.42,1.30]	0.76 [0.50,1.17]
**Hispanic**	1.52 [0.62,3.74]	1.54 [0.72,3.31]	0.63 [0.37,1.06]	1.33 [0.74,2.37]
**Race**				
(White)	-	-	-	-
African-American	0.84 [0.48,1.45]	1.06 [0.62,1.82]	0.75 [0.45,1.24]	0.84 [0.50,1.43]
Other	1.03 [0.54,1.93]	1.77 [0.85,3.69]	1.26 [0.67,2.39]	0.91 [0.49,1.69]
**Income**				
< $25,000	2.04* [1.04,4.02]	1.68 [0.84,3.36]	2.07* [1.14,3.76]	2.33* [1.16,4.69]
$25,001–50,000	1.98* [1.17,3.34]	0.98 [0.56,1.69]	2.00* [1.16,3.46]	1.64 [0.90,2.98]
$50,001–75,000	1.25 [0.70,2.25]	0.99 [0.57,1.69]	1.84* [1.11,3.04]	1.05 [0.58,1.91]
(> $75,000)	-	-	-	-
**Externalizing behaviors**	1.01 [0.90,1.13]	0.99 [0.89,1.10]	0.98 [0.88,1.09]	1.03 [0.95,1.13]
**How well youth converses**				
(No trouble)	-	-	-	-
A little trouble	0.84 [0.39,1.80]	1.08 [0.56,2.07]	1.91* [1.01,3.59]	2.08* [1.10,3.97]
A lot of trouble	1.15 [0.52,2.55]	3.05** [1.56,5.94]	1.68 [0.84,3.39]	2.29* [1.19,4.42]
Not at all	0.98 [0.32,2.98]	10.29*** [4.03,26.28]	2.13 [0.82,5.52]	4.17*** [1.83,9.50]
**Social communication**	0.53*** [0.46,0.62]	0.72*** [0.64,0.82]	0.64*** [0.57,0.73]	0.93 [0.84,1.02]
**Functional cognitive skills**	0.97 [0.90,1.05]	0.84*** [0.78,0.90]	0.93* [0.87,0.99]	0.89** [0.83,0.96]
**ADHD**	0.92 [0.60,1.41]	0.83 [0.53,1.32]	0.81 [0.54,1.22]	0.83 [0.53,1.29]
**Type of school**				
(Regular)	-	-	-	-
Special	1.43 [0.75,2.74]	1.25 [0.55,2.82]	1.34 [0.69,2.60]	1.32 [0.69,2.55]
Other	0.32 [0.08,1.33]	0.65 [0.22,1.92]	0.51 [0.19,1.35]	0.55 [0.16,1.93]
**School size (per 100 children)**	1.00 [0.97,1.03]	0.99 [0.96,1.02]	1.00 [0.97,1.02]	1.02 [0.99,1.05]
**Student spends any part of day in special classroom**	1.60 [0.66,3.88]	1.25 [0.61,2.57]	1.38 [0.68,2.82]	0.65 [0.30,1.41]

Source: National Longitudinal Transition Study 2.

Number of multiply imputed data sets  = 50. Weighted to population levels. Variances adjusted for sampling method.

## Results

Compared with those from other groups (Table 2), adolescents with an ASD were more likely to be male (84.5%), more concentrated in the highest income category (23.0% with family income > $75,000) and less concentrated in the lowest income category (22.0% with family income < $25,000), more conversationally impaired (adolescents with an ASD were 3–4 times more likely to be in the two lowest levels of conversational ability), and more likely to attend a special school (10.4% vs. 3.6% for MR, the next highest group). Hispanic representation was similar for ASD (11.0%) and MR (10.9%) but lower than SP (19.1%) and LD (20.8%).

Adolescents with an ASD were significantly more likely never to see friends (43.3%), never get called by friends (54.4%), or never be invited to activities (50.4%) compared with adolescents from all the other groups (Table 3). These adolescents also had significantly lower rates of participation than adolescents with LD or SP for all measures of general social participation except taking lessons outside of school and belonging to a performing group. There were two significant differences in general social participation between adolescents with an ASD and those with a primary label of MR: those with an ASD were more likely to take lessons outside of school (30.6% vs. 19.6%) and less likely to belong to a sports team (16.3% vs. 22.3%). Adolescents with an ASD were significantly more likely to belong to a group that included primarily adolescents with special needs (24.9%) than adolescents from all other groups.

Table 4 reports rates (as percentages) of four indicators of limited social participation among adolescents with an ASD (never sees friends, friends never call, never invited to activities, and no extracurricular activities) stratified by the covariates. Table 5 uses logistic regression to examine the adjusted association between each of these four outcomes and the same set of covariates. Adolescents with an ASD from families in all three lower income groupings had significantly higher odds of never being invited to activities relative to those from families with incomes > $75,000 (Table 5). Adolescents with an ASD from families with income < $25,000 had significantly higher odds of no involvement in any extracurricular activities compared with those from families with incomes > $75,000. Those from families with income < $50,001 had significantly higher odds of never seeing friends compared with those from families with incomes > $75,000. Conversational impairment was associated with higher odds of friends never calling, never being invited to activities, and having no involvement in extracurricular activities. Higher social communication ability was significantly associated with lower odds of never seeing friends, friends never calling, and never being invited to activities. Higher functional cognitive skills were significantly associated with lower odds of friends never calling, never being invited to activities, and no extracurricular activities.

## Discussion

Our findings using a nationally-representative sample of adolescents with an ASD indicate that half experience no or very limited social activities with friends and only one-third participate in social activities in the community with peers. These participation rates were significantly lower than those observed in three other disability groups: adolescents with speech/language impairments, learning disabilities, and mental retardation.

Rates of social activities with groups were lower than reported personal interactions with friends. Whereas about half of the adolescents with an ASD got together with friends, received phone calls, and were invited to activities by friends, only one-third participated in group social activities. They were most likely to volunteer or provide community service (35.1%) or take lessons or classes outside of school (30.6%). In terms of belonging to community groups, about one-quarter belonged to a religious group and the same number belonged to a disability specific group. Fewer belonged to sports teams or performing groups.

Overall, these findings show that the majority of adolescents with an ASD experience major obstacles to social participation. It appears that experiences with peers are more likely to occur one on one, and perhaps at home rather than in the community. One mechanism for promoting social relationships is by fostering participation with peers in group activities. With only one-third of adolescents with an ASD accessing such opportunities, there is an obvious need for greater supports and services to promote community inclusion for this population.

Social participation with friends was the factor that most differentiated adolescents with an ASD from those in the other three disability groups included in the study. Those with an ASD had fewer experiences with their friends outside of school and were three to five times more likely never to get together with friends compared with peers from all three other disability groups. These findings concur with those of Solish et al., who found that half of their sample of children and adolescents with an ASD had no friends, compared with less than one-quarter of adolescents with an intellectual disability (mental retardation) [17].

The one notable exception to the lower rates of community participation by adolescents with an ASD was their higher rate of participation in disability-related groups. These findings suggest that the extracurricular activities of adolescents with an ASD may frequently take place in non-inclusive settings. Our data cannot answer whether this disproportionate rate of participation is driven by the motivations and choices of adolescents or parents or some other factor. Understanding the dynamics underlying this disproportionate rate of participation could be deepened by mixed methods studies involving a qualitative component.

Not surprisingly, conversational impairment and low social communication skills were associated with a lower likelihood of social participation. Having impaired conversational ability was associated with an elevated risk for friends never calling, never being invited to activities, and having no involvement in extracurricular activities. Having a higher social communication score (based on questions about joining groups, making friends, social confidence, and conversation initiation) was associated with lower odds of never seeing friends, friends never calling, and never being invited to activities. This is consistent with prior work which found a lower likelihood of friendships among those with an ASD if they scored poorly on an ADI-R item about social impairment [18]. Our findings highlight the centrality of social communication abilities and suggests these adolescents continue to need the kind of supports typically provided by speech/language pathologists. Research into effective ways of promoting social communication abilities should be prioritized if we want to increase the social success of adolescents with an ASD.

After controlling for other variables, we found several notable correlates of four measures of limited social participation among adolescents with an ASD. Two of the measures were about friendships (never sees friends, friends never call) and two were about activity participation (never invited to social activities, having no extracurricular activities). Adolescents from families with lower income had an elevated risk for no involvement in activities, but not an elevated risk for limited contact with friends. This is consistent with prior research using NLTS2 data on all high school students enrolled in special education (i.e. not broken out by disability category) that youth involvement in extracurricular activities is significantly more likely among wealthier families [19]. In contrast, this prior research found that students from higher-income families had a significantly lower likelihood of never seeing friends. The correlation of income and participation in our findings clearly suggests that future research should include better measures of access related to social activity.

Higher functional cognitive skills were associated with a lower risk for limited social participation across all four measures. This is consistent with the finding of Mazurek and Kanne that children and adolescents with an ASD with IQ<85 were less likely to have friendships as measured by a single ADI-R item [6]. Again, our work extends prior research by pointing to the pervasive association between individual abilities and a wide range of social participation indicators.

Notably, having neither comorbid ADHD nor high externalizing behaviors was significantly associated with any indicators of social participation. Difficulties with friendships and social participation are well documented among children with ADHD and those with externalizing behaviors [20]. Our lack of confirmation could be due to using a measure of externalizing behaviors that is weak on reliability and validity.

Age, sex, race, ethnicity, and school context factors were not significantly related to social participation outcomes in the multivariate models. In results not reported, we examined a variety of indicators of inclusion in general education settings beyond the one we entered in our final regression model (whether the student spends any part of their day in a special education classroom). None were significantly associated with social participation indicators in multivariate models that controlled for other factors. This is consistent with our prior work, which did not find an association between regular education inclusion and friendships for adolescents with an ASD [18].

Several recent reviews have summarized findings about intervening to improve social skills in children with an ASD [21], [22]. As in most areas of research on ASDs, much less is known about how to intervene with adolescents. Our findings simultaneously underscore the fact that impairments in social communication are a core challenge for adolescents with an ASD and that these core challenges are strongly associated with a wide range of social participation outcomes. Some research suggests that social communication skills can be improved through targeted intervention. However, successful generalization of skills remains a substantial challenge. Improving social communication skills may not automatically result in increased social participation. Our findings suggest it is also important to look at the family socioeconomic context and how that may affect access to social opportunities.

Inclusion in the NLTS2 sample was based on schools' determination of meeting eligibility criteria for special education services under the autism category. Strictly speaking, our findings generalize to adolescents with an ASD who are enrolled in the special education autism category rather than to all adolescents with an ASD. How representative of all adolescents with an ASD are our results? We cannot answer this definitively. The male:female ratio of 6.5:1 in this ASD special education population is slightly higher than the mean of 4.5:1 across recent epidemiological surveillance site estimates but still within the range of prior research [1], [13].The distribution by race and ethnicity in this study was similar to population-based surveillance findings [23]. The higher rate of conversational impairment relative to adolescents from other eligibility groups is consistent with the fact communicative impairment is a hallmark diagnostic feature of ASDs. Household income among adolescents with an ASD in this study tended to be higher relative to those from other special education categories. This is consistent with other research indicating that autism is identified at a higher rate among more affluent families, suggesting that poorer children with autism are commonly under-identified [24]. Lower household income was associated with limited social participation in our study. If our sample under-identifies the true prevalence of ASD among lower income households then the income association may be biased.

Overall, our findings suggest that a mix of individual and socioeconomic factors can significantly influence a range of social participation outcomes. What are the implications for intervention and policy? At the broadest level of interpretation, there is a need for researchers and practitioners to focus on expanding opportunities for social participation and on improving individual social competence. This conclusion is consistent with the conceptual model of disability put forth in the World Health Organization's International Classification of Functioning, Disability, and Health [25]. This landmark document challenges a purely clinical focus on treating individual impairments by depicting the outcomes of disability as a function of both individual and contextual factors.

Our study has some limitations. The survey-based measures of social participation were not as fine-grained as in some other studies and were reported by parents. Furthermore, the available survey measures were focused on the quantity of social participation rather than the quality. It is possible that the quality of social connections is more important than the number. We did not have information about the size of each youth's social network. Our study also lacked a comparison group of typically developing peers.

Our study also has several important strengths. The external validity of our findings was greatly enhanced by the representativeness of the sampling strategy and the size and diversity of the sample. We were able to test hypotheses about race, ethnicity, and socioeconomic status which are often neglected in ASD research. Finally, the availability of comparison groups from other special education categories enhanced our ability to contextualize findings.

In summary, compared with prior research, our study significantly expands inquiry in this area by broadening the range of social participation indicators examined, by increasing the external validity of findings, by focusing on the under-studied developmental stage of adolescence, and by taking an ecological approach that included many potential correlates of social participation. Future research needs to extend this inquiry into adulthood while also looking at individual and contextual characteristics, such as access, which may influence social participation. Finally, research in this area needs to catch up with the rapidly changing landscape of normative social participation by developing measures of new forms of electronically mediated social interaction, by broadening intervention goals to include helping adolescents with an ASD participate in these new forms of interaction, and by considering how to take advantage of a prevalent proclivity for computer use to see how electronic media might become a platform for delivering effective social competence interventions.
